# INVESTIGATING DREAMING IN COGNITIVELY DIVERSE OLDER ADULTS

**DOI:** 10.1093/geroni/igad104.3441

**Published:** 2023-12-21

**Authors:** Warren Britton, Jessica Strong

**Affiliations:** University of Prince Edward Island, Charlottetown, Prince Edward Island, Canada; University of Prince Edward Island, Charlottetown, Prince Edward Island, Canada

## Abstract

Dreaming changes across the lifespan. While our understanding of how dreaming relates to aging and well-being has developed with respect to early and middle phases of the lifespan, the dreams of older adults remain under-studied. Reviews of the literature suggest decreases in dream recall and the emotionality of dream content that often precede age-related changes in sleep architecture, with few changes in dreaming occurring between ages 45-75, after which dream recall declines steeply. However, these findings are based on relatively few studies. This point is underscored when considering the effects of secondary aging on dreaming, where virtually no literature exists on the dreams of older adults experiencing mild cognitive impairment. Studying the dreams of older adults experiencing cognitive impairment may seem trivial when compared to other processes implicated in secondary aging, but recent studies examining the relationship between frequency of negative dream content and the increased risk of onset and acceleration of cognitive decline beg a closer examination of how dreaming contributes to a broader understanding of health and functioning across the lifespan. The scoping review of sleep, dreams, well-being and cognition across the lifespan, and the subsequent proposed study design seeks to employ the Hall and Van de Castle Coding System of content analysis to contribute to the literature on the dream frequency and content of cognitively typical older adults while also exploring the feasibility and methodological considerations of studying the dream content of older adults with mild cognitive impairment.

